# Social information about others' affective states in a human‐altered world

**DOI:** 10.1111/1365-2656.70208

**Published:** 2026-01-20

**Authors:** Luca G. Hahn, Jordan McDowall, Margaux Vanhussel, Mike Mendl, Alex Thornton

**Affiliations:** ^1^ University of Exeter Penryn UK; ^2^ Swansea University Swansea UK; ^3^ University of Bristol Bristol UK; ^4^ University of Liège Liège Belgium

**Keywords:** affective states, behavioural flexibility, cognition, conservation, environmental change, social information, uncertainty, welfare

## Abstract

1. As a result of human‐induced environmental change, animals increasingly face challenges that differ from those encountered throughout their evolutionary history. While this has caused dramatic declines for many species, some can persist by gathering information to reduce uncertainty, thereby minimising risks and exploiting new opportunities. The strategic use of social information can be particularly useful in enabling such uncertainty reduction.
2. Here, we argue that the behavioural and affective states of others provide vital social information for animals to guide evaluations of risks and opportunities. Specifically, attending and responding to indicators of others' affective states through processes such as emotional contagion may facilitate information transmission. For instance, when exposed to a novel, ambiguous anthropogenic stimulus that could indicate either an opportunity or a threat, animals may use social information about others' affective states to decide whether to approach or avoid the stimulus.
3. To increase immediate and long‐term benefits, individuals might also alter their social behaviour and information use flexibly based on critical early‐life experiences, the socio‐ecological context or the behaviour and states of associates in the social network.
4. Finally, given that an individual's affective state can influence how it copes with changing environments and makes appropriate decisions, we argue that there is a need for greater synergy between animal welfare and conservation efforts. Bridging the gap between ensuring individual‐level welfare and population‐level resilience will be crucial for ethical policies to protect wild animals responsibly in the face of human‐induced rapid environmental change.

## Graphical abstract

Faced with anthropogenic change, animals now encounter challenges different from their evolutionary past. To cope with such challenges, animals may use social information about others' affective states to guide their decisions. Considering affective states of wild animals could have important implications for animal welfare and wildlife conservation.

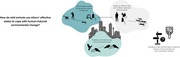

## 1 INTRODUCTION

Despite the unprecedented challenges posed by the rapid pace of human‐induced environmental changes, some animals can persist and even thrive in human‐altered environments. As evolutionary change through natural selection is often too slow to enable adaptation, these animals seem to cope with anthropogenic change due to their high behavioural flexibility (Vardi & Berger‐Tal, 2022). Often referred to as ‘urban adapters’ and ‘exploiters’ (see Glossary for definitions of key terms), these species can take advantage of environments with varying levels of human disturbance by altering aspects of their behaviour (McKinney, 2006). For instance, a comparative study on wild boar (*Sus scrofa*) populations showed that urban individuals' diets contained a higher proportion of anthropogenic foods, suggesting that changes in feeding behaviour supported the exploitation of urban niches (Castillo‐Contreras et al., 2021; Sih et al., 2011). Although there is growing evidence that many different species can benefit from exploiting novel resources in urban environments (Sih et al., 2011), human activities can also generate substantial uncertainty, which may require animals to attend and respond to information to make adaptive decisions (Lee & Thornton, 2021). Uncertainty, a concept from information theory (Shannon, 1948), is considered high when different outcomes of variables, such as external stimuli or an individual's actions, are equally likely or useful. Thus, uncertainty can be high if an animal is faced with an ambiguous anthropogenic stimulus, such as a novel object, that could indicate a threat and/or an opportunity. The reduction of such uncertainty could be achieved through different mechanisms, potentially involving cognition (Griffin et al., 2017; Lee & Thornton, 2021) and affective states, and recent theories propose that uncertainty reduction is a key function of the brain (Friston, 2010).

Although they are often considered separately, cognition and affective states are likely complementary and closely linked mechanistically and functionally in resolving uncertainty and driving decision‐making in animals (Pessoa, 2008). Cognition can broadly be defined as the neural processes that involve gathering, processing, storing and acting upon information from the environment (Shettleworth, 2010), and the role of cognition in coping with environmental change has sometimes been referred to as a ‘cognitive buffer’ (Sol, 2009a, 2009b). Information that is processed cognitively can be obtained individually (personal information), or from other individuals, such as conspecifics and heterospecifics (social information) (Danchin et al., 2004). Affective states are also an important mechanism through which animals evaluate their environment and make decisions (Mendl & Paul, 2020). While there is no ubiquitous definition of affective states, we define them as short‐ and long‐term mental states which are valanced: that is, they are positive or negative; pleasant or unpleasant (Mendl & Paul, 2020; Russell, 2003). This definition stems from our own conscious experiences of mental states (‘feelings’) that we label as emotions or moods (Mendl et al., 2022). Because we cannot directly measure subjective states in non‐human animals (we use language as a gold standard, yet fallible, measure in humans), we cannot be certain about whether and which other species consciously experience them; hence, this issue remains a topic of heated debate (e.g. Boly et al., 2013; Klein & Barron, 2016; Panksepp, 2005; Paul et al., 2020). Nevertheless, by considering affective states as comprising components including subjective, behavioural, physiological, neurological and cognitive changes (Paul et al., 2005; Scherer, 1984), it is possible to scientifically study animal affect in the absence of certainty about the conscious subjective component, by measuring the other components (Mendl et al., 2022). Thus, indicators of affective states in response to environmental stimuli, such as anthropogenic stimuli, can be objectively measured through physiological, neurological, behavioural and cognitive markers (Mendl & Paul, 2020).

**FIGURE 1:**
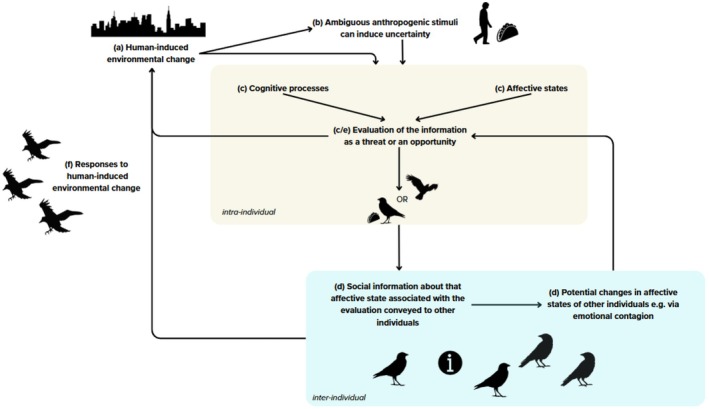
Human‐induced environmental changes (a) can generate uncertain situations confronting animals with ambiguous anthropogenic stimuli (b) that will elicit cognitive and affective responses based on both internal and external cues (c/e). Those responses may entail social information that can influence others' states and responses (d). By acting upon such cues, animals can—on the long term—shift their behaviour, physiology or ecology, which can have an influence on the human‐induced environmental changes themselves (e.g. abandon a site previously occupied; Carrete et al., 2007) (f). Mental states of animals can play a significant role in how they respond to human‐induced anthropogenic change. Both cognitive processes and affective states can influence how an animal processes, evaluates and acts upon information about external anthropogenic stimuli. In some cases, animals' responses to human‐induced change may also feedback to influence human behaviour. For example, in Sydney, Australia, wild sulphur‐crested cockatoos (*Cacatua galerita*) have learned to open bins to access food, which led humans to respond with countermeasures. This could potentially lead to an ‘innovation arms race’ between cockatoos and humans (Klump et al., 2022). Jackdaw silhouettes in (d) are from *Phylopic* (uploaded by Birgit Lang and Ferran Sayol).

Anthropogenic activities may pose uncertainty that could induce and influence measurable cognitive, behavioural and affective responses in animals (Anderson et al., 2019) (Figure 1). For example, urban herring gulls (*Larus argentatus*) show similar behavioural and affective responses to conspecific alarm calls and human shouting (Di Giovanni et al., 2022). Human shouting induces uncertainty here because it may correspond to a human threatening the gull, or the shouting may be unrelated to the gull's presence and thus would not pose a threat. In animals including humans, uncertainty tends to induce a negative affective state, such as discomfort and distress (although positive affective states are also possible) (Anderson et al., 2019; Grupe & Nitschke, 2013). Such a negative affective state may, in turn, serve as a mechanism eliciting adaptive responses to resolve such uncertainty, for example, by driving the animal to gather additional information about a stimulus (thus alleviating the affective state of discomfort). Therefore, affective states can be an important mechanism driving decision‐making in animals (Mendl & Paul, 2020), and as such, should be considered to better understand the decisions that animals make when confronted with anthropogenic change.

Here, we argue that the ability to utilise social information about the affective states of others, a common ability in many animals, may play a significant role in enabling adaptive behavioural flexibility and could thus be an important mechanism driving decision‐making in animals (Mendl & Paul, 2020) faced with anthropogenic change. For instance, a study conducted on bottlenose dolphins (*Tursiops truncatus*) showed that individuals that performed synchronous swimming at a higher rate (an affiliative behaviour) were more likely to respond to ambiguous cues as predicting a positive rather than less positive outcome (Clegg et al., 2017). Indeed, Clegg et al. (2017) reasoned that more affiliative behaviour may cause or be caused by a more positive affective state, which could also act to buffer stress in response to uncertainty. The ability to process information about their own and others' affective states, such as through affiliative behaviour, may allow some animals to respond appropriately in uncertain situations by distinguishing between the likelihood of a situation or stimulus predicting a threat as opposed to an opportunity: a vital skill in human‐altered environments.

To cope with human‐altered environments through social information use, animals may integrate cognitive and affective processes (Figure 1c). One cognitive process, social learning, which can be defined as ‘learning that is influenced by observation of, or interaction with, another animal (typically a conspecific) or its products’ (Heyes, 1994), may have an inherently affective component (Gruber et al., 2021). For instance, an affective component in social learning could enable transfer of information about the value of stimuli and serve as feedback from the demonstrator to the learner (Gruber et al., 2021). If affective states become salient to conspecifics, for example, via cues or signals such as vocalisations (Briefer, 2018) or facial expressions (Parr et al., 2009), then individuals may use others' affective states as a source of social information (Van Kleef, 2009) to reduce uncertainty and to make adaptive decisions in human‐altered environments. One could argue that it is sufficient for animals to use the overt behaviours of other individuals performing a task (e.g. approaching a novel object or food item) as sources of information without the need to attend or respond to indicators of affective states. Although this may often be the case, we argue that attention to (potentially subtle) cues of others' affective states may provide additional, more fine‐scaled social information about stimuli and outcomes in the environment. Attending to this aspect of *how* a task is performed is potentially more beneficial than just attending to the main elements of task performance. For example, an individual may observe a conspecific approaching a novel food item while expressing behaviour indicative of fear‐ or disgust‐like affective states. This has been found in great tits (*Parus major*), which, after observing a conspecific consuming a visually recognisable food source and expressing visual aversion signals such as dropping seeds and beak‐wiping, subsequently showed a significant aversion to that specific food item (Landová et al., 2017). Here, dropping seeds is directly related to task performance, whereas beak‐wiping is more likely to be related to the animal's affective state, expressing a subtle yet noticeable cue of the individual's discomfort. Information about affective states may thus provide more salient, fine‐scale information about the potential opportunities and risks associated with a stimulus as compared to simply observing the conspecific approach the food item. As affective states often manifest through behaviours, animals are likely to use behavioural indicators as sources of social information. Attending to overt behaviours is thus a prerequisite for the ability to respond to affective states.

Our understanding of whether affective states could help animals to cope with anthropogenic change is currently very limited. Indeed, while there is growing evidence from laboratory studies that affective states influence decision‐making (Harding et al., 2004; Mendl & Paul, 2020), affective states are seldom considered in the context of environmental change, particularly in the wild (Crump, 2021). In this opinion piece, we address this gap by considering how social information about conspecifics' affective states may help wild animals navigate human‐altered environments by reducing uncertainty about opportunities and threats (Oliveira & Faustino, 2017). In Section 2, we ground our arguments in evolutionary theory and behavioural ecology to discuss in more detail how animals may use and benefit from (transmission of) information about their own and others' affective states to guide decision‐making in response to anthropogenic environmental change. In Section 3, we end by examining the potential implications and applications for (i) individual‐level animal welfare and (ii) population‐ and species‐level conservation. While these two perspectives are typically considered separately, we argue that there are important benefits to applying them together, with short‐term indicators of affective states potentially informing long‐term conservation measures.

## 2 SOCIAL INFORMATION ABOUT AFFECTIVE STATES CAN GUIDE ANIMALS' DECISIONS IN A HUMAN‐ALTERED WORLD

### 2.1 Affective states as a source of information and a mechanism for decision‐making in animals

The behavioural responses of animals to human disturbances are well documented (e.g. Lott & McCoy, 1995; Sih, 2013; Treves & Brandon, 2005), but less attention has been given to how animals appraise these changes via changes in their own affective states and those of others. Humans' presence, whether direct, through activities such as tourism and outdoor sports, or more indirect, for example, through habitat destruction or pollution, can elicit and influence a range of indicators of affective states in animals such as behavioural, physiological or cognitive components (Crump, 2021). These include markers of positive states as in excitement, joy or relief after avoiding a negative outcome, as well as of negative states like fear, anxiety or frustration (Goumas et al., 2022; Mendl & Paul, 2020; Nelson et al., 2023). A clear example of a direct influence of human activities on affective states is seen in the artificial feeding zones established for macaques in tourist‐heavy temple areas. A study on wild male Barbary macaques (*Macaca sylvanus*) in Morocco found a strong positive correlation between the frequency of aggressive encounters with tourists and an increase in self‐scratching behaviour—a well‐established indicator of anxiety (Castles et al., 1999; Maestripieri et al., 1992)—as well as elevated faecal glucocorticoid (fGC) levels during the interactions (Maréchal et al., 2011). By contrast, human‐induced environmental change may also impact affective states of wild animals more indirectly. Habitat destruction can influence local population density, which in turn affects the likelihood, intensity and outcomes of aggressive interactions among conspecifics, as well as foraging effort (see Fisher et al. (2021), for how environmental change may impact social interactions). A relevant example comes from a study on ring‐tailed lemurs (*Lemur catta*) in two fragmented forests in Madagascar. Gabriel et al. (2018) found that the population with the highest individual density exhibited elevated fGC metabolite concentrations. These increased stress hormone levels were associated with behavioural factors such as foraging effort, intergroup encounter rate and intragroup agonism, suggesting heightened social stress due to habitat reduction. However, glucocorticoid levels alone may not be reliable indicators of affective valence (Buwalda et al., 2012). Understanding how animals appraise and respond to human‐induced changes through affective states is therefore essential for assessing the broader consequences of anthropogenic activities on animal behaviour and decision‐making.

Animals can use their own and others' affective states as a heuristic and source of information to make decisions (Mendl & Paul, 2020). ‘Optimistic’ or ‘pessimistic’ judgements about ambiguous situations, as mentioned earlier for dolphins, are hypothesised to be linked to background affective state with animals in a more negative state being predicted to show more ‘pessimistic’ decisions (Mendl et al., 2010; Mendl & Paul, 2020). This may have adaptive value, and hence cross‐species generality, given that threatening environments are likely to generate negative affective states which can then, in turn, be used by the animal as a heuristic, or Bayesian prior, indicating elevated likelihood of dangerous outcomes and thus promoting cautious (e.g. ‘pessimistic’) decisions. Such judgement biases have been studied by training animals that one cue predicts a positive outcome (e.g. food) which can be obtained by performing one type of response, while a different cue predicts a negative outcome (e.g. no food; noise) that can be avoided using a different type of response (Harding et al., 2004). Ambiguous cues that are intermediate between the training cues are then occasionally presented to see whether the animal demonstrates the response predicting the positive (‘optimistic’) or negative (‘pessimistic’) outcome. Studies indicate that, as predicted, animals assumed to be in more positive states generally show more ‘optimistic’ judgement biases (Neville et al., 2020) and therefore that these biases can be a valuable cognitive marker of animal affective states.

Affective states may be coupled with and solidify the process of learning associations between stimuli and their outcomes, for instance in the case of fear learning (Olsson & Phelps, 2007). For example, mobbing or alarm responses of conspecifics, which may reflect and induce negative affective states in mobbers (as described in the example on herring gulls above; Di Giovanni et al., 2022), may be sufficient for some animals, such as blackbirds (*Turdus merula*), jackdaws (*Corvus monedula*) and American crows (*Corvus brachyrhynchos*) to learn to avoid novel heterospecifics (Cornell et al., 2012; Curio et al., 1978; Lee et al., 2019). This may facilitate adaptive responses that allow animals to avoid a novel, potentially threatening situation. Thus, indicators of affective states in others could be used as a way of summarising information about the environment. Indeed, there is growing evidence indicating that animals, particularly vertebrates from primates to rodents to domestic animals such as horses and dogs, are able to recognise affective states in other individuals by using and integrating different sensory modalities (reviewed by Ferretti & Papaleo, 2019). For instance, the use of facial expressions to convey and extract information about affective states has been demonstrated across different mammalian taxa, such as different primates and sheep (Tate et al., 2006), and may also be relevant in birds (Arnould et al., 2024; Bertin et al., 2018). Sheep showed an untrained preference for images of the faces of ‘calm’ conspecifics (i.e. photos taken in an assumed ‘calm’ context) over face‐pictures of ‘stressed’ conspecifics, with similar preferences observed for images of smiling compared to angry human faces (Tate et al., 2006). These findings indicate that sheep can respond in an appropriate way (e.g. approach vs. avoid) to facial expressions associated with affective states in both conspecifics and humans. A study by Albuquerque et al. (2016) found that dogs attended longer to facial expressions associated with play or aggression when these expressions were accompanied by a *congruent* vocalisation compared to when the accompanying vocalisation was associated with a different facial expression. This suggests that dogs can integrate auditory and visual information associated with specific affective states and hence may have at least prototypical emotion concepts. These inferences about the detection and use of affective information from other conspecifics are supported by evolutionarily conserved brain structures and neuronal networks responsible for emotion recognition, and neuroimaging studies (Tate et al., 2006). Other examples concern different aspects of body language and postures that may reflect affective states (Guesgen & Bench, 2017), such as changes in movement (e.g. freezing and other anti‐predator behaviour) (Roelofs, 2017) that are observed across a range of taxa, from birds (Papini et al., 2019) to fish (Oliveira et al., 2017) to mammals (Reimert et al., 2013). Furthermore, as seen in the dog example, vocalisations are often thought to be salient and informative indicators of the caller's affective state, especially in mammals and birds (Briefer, 2018).

The extent to which individuals attend and respond to others' affective states may be modulated by factors such as relatedness, familiarity and affiliation. Moreover, the ability to recognise others' affective states may be particularly relevant in changing, uncertain environments and social information may be particularly useful under such circumstances (as seen in the use of ‘copy when uncertain’ social learning strategies—Laland, 2004). For example, bumblebees (*Bombus terrestris*) relied more on social learning when rewards were highly variable (i.e. more uncertain) than when they were not (Smolla et al., 2016). In the case of the example on Barbary macaques mentioned above, using social information about the self‐scratching behaviour indicating anxiety of other individuals may allow observers to reduce uncertainty about the situation and may cause them to become more alert, and thus potentially avoid danger.

### 2.2 Animals can influence each other's affective states and decision‐making in a human‐altered world

Not only do animals use social information about others' affective states to make decisions, but perceiving another's state may lead to its direct transmission through emotional contagion: defined as the matching of affective states among individuals (Figure 1; Meyza et al., 2017; see Dezecache et al., 2015; Pérez‐Manrique & Gomila, 2022 for more comprehensive reviews about emotional contagion in animals). This phenomenon (Pérez‐Manrique & Gomila, 2022) can propagate positive and negative affective states within dyads and groups and is therefore of particular importance for the transmission of social information. Emotional contagion can be underpinned by different mechanisms and sensory modalities. For emotional contagion to arise, animals may use and be influenced by different visual, auditory, olfactory and tactile, indicators of affective states, and may also integrate information across modalities (Pérez‐Manrique & Gomila, 2022). For instance, animals may use and be influenced by information obtained from visual stimuli, such as facial expressions (Palagi et al., 2020) or body language (e.g. self‐scratching reflecting anxiety) (Castles et al., 1999) but also auditory stimuli, such as the acoustic features of calls (Briefer, 2018). There is behavioural and neurophysiological evidence to suggest that emotional contagion is widespread among vertebrates (Pérez‐Manrique & Gomila, 2022), with empirical support for its occurrence in birds (Edgar & Nicol, 2018; Wenig et al., 2021), fish (Burbano Lombana et al., 2021; Kareklas & Oliveira, 2024) and mammals (Huber et al., 2017; Keysers et al., 2022). For instance, individuals who did not encounter the stimulus inducing a negative affective state themselves may still exhibit a comparable affective state by interacting with those who did face such a stimulus (Adriaense et al., 2019; Oliveira et al., 2017). One empirical approach to examine affective states indicative of emotional contagion is the judgement bias approach discussed above. For example, Adriaense et al. (2019) demonstrated that ravens observing a conspecific in an induced negative state showed a ‘pessimistic’ judgement bias, indicating that the expressive behaviour of the demonstrator bird influenced the affective state of the observer—an example of emotional contagion.

Currently, little is known about how widespread emotional contagion is across taxonomic groups (even within vertebrates, such as amphibians and reptiles). Further research, using standardised and comparable protocols to assess affective states and their transmission, will be crucial for advancing our understanding of this phenomenon across the animal kingdom. Though this work is still in its infancy, two main lines of evidence lead us to hypothesise that emotional contagion could be more widespread across the animal kingdom than has thus far been demonstrated. First, there is a growing body of evidence that affective processes may occur in invertebrates such as insects or molluscs that were previously thought to lack them (Bateson et al., 2011; Birch et al., 2021; Perry & Baciadonna, 2017). Second, social information use has been shown to be extremely widespread, even in species (e.g. tortoises or non‐colonial crickets) generally seen to be relatively asocial (Webster, 2023). Thus, it does not seem unreasonable to speculate that the affective states of others provide a valuable source of information (e.g. see Romero‐ González et al. (2025) for recent evidence of positive emotional contagion in bumblebees).

Emotional contagion can have implications for the welfare of animals (Špinka, 2012). Consequently, investigations into emotional contagion have focused extensively on social animals in captive conditions, particularly in relation to empathy: defined as an affective response to the affective state of another individual (De Waal, 2008; Preston & de Waal, 2002). For example, early studies demonstrated that rats exhibited a fear‐like state (characterised by a reluctance to press a lever) in response to distressed conspecifics, while more recent works show that rats free conspecifics from restraint, even when given the alternative of a food reward (Ben‐Ami Bartal et al., 2011; Church, 1959), suggesting an empathetic response to a conspecific's plight (but see Silberberg et al., 2014). Using a judgement bias approach, Adriaense et al. (2019) showed that common ravens (*Corvus corax*) observing conspecifics in an induced negative affective state showed ‘pessimistic’ responses to ambiguous cues indicating emotional contagion from demonstrator to observer. Emotional contagion has been thought to be the biological basis of empathy, with the latter requiring additional processes related to theory of mind, that is reasoning about others' mental states (De Waal, 2008). Distinguishing emotional empathy from emotional contagion (e.g. behavioural and physiological matching) remains a challenging enterprise (Edgar, Nicol, et al., 2012). Nevertheless, both emotional contagion and empathy‐like states may have adaptive value in facilitating efficient transfer of information, for example, about threats and opportunities. This could unfold via processes such as social buffering and social stress transmission, which we discuss below.

Social buffering and social stress transmission could be viewed as two complementary forms of emotional contagion and information transmission (Brandl et al., 2022; Kikusui et al., 2006; Oliveira & Faustino, 2017). Social buffering occurs when social support provided by social partners attenuates stress responses (Kikusui et al., 2006). This process can occur without consolation (such as physical touch) from a conspecific: that is, simply the presence of a close affiliate is sufficient in eliciting a calming effect (Kikusui et al., 2006). Social buffering has been reported in multiple species and can be mediated through visual, vocal and olfactory signals (Kiyokawa & Takeuchi, 2017; Peirce et al., 2000; Rukstalis & French, 2005). For example, wild chimpanzees exhibited lower urinary glucocorticoid levels in response to a natural stressor (such as inter‐group encounters) in situations in which they were accompanied by a bonded partner compared to when they were with non‐bonded individuals (Wittig et al., 2016). Conversely, social transmission of stress occurs when the state of distress of individuals can elicit a stress response in others (Brandl et al., 2022). For example, when in colonies with stress‐exposed individuals, non‐stressed exposed zebra finches (*Taeniopygia guttata*) reduced their movement and strengthened their pair‐bonding behaviour but maintained fewer relationships with other group members, indicating stress transmission (Brandl & Farine, 2024). These processes of emotional contagion can also be conceived as a transmission of social information to reduce uncertainty about a given situation, such as to evaluate a potential threat (Oliveira & Faustino, 2017). Uncertainty may arise when an individual is confronted with a situation that could equally likely pose an opportunity and a threat; for instance, whether it is best to approach or retreat from a novel stimulus. Individuals may use social information to compare their own to others' affective states and adjust their behaviour accordingly (Oliveira & Faustino, 2017). An individual may experience fear when faced with a novel stimulus, but other individuals' behaviour may indicate the absence of danger—this social information about others' states could then lead to social buffering of the distressed individual's affective state. For example, wild meerkat (*Suricatta suricatta*) pups were more likely to incorporate novel foods into their diet if they had previously interacted with experienced adults consuming those foods (Thornton, 2008). The use of social information during decision‐making under uncertainty has been shown to increase true positives and decrease false positives, thus enhancing the accuracy of decisions (Wolf et al., 2013). The efficiency of emotional contagion (i.e. social buffering and social stress transmission) may itself be influenced by anthropogenic change. For instance, noise pollution could impact the effectiveness of vocal signals mediating social buffering or the transmission of stress responses (Wong & Candolin, 2024).

### 2.3 Flexibility in social information use about affective sates to cope with environmental change

In the face of rapid human environmental change, using social information about others' affective states may allow animals to exhibit and enhance behavioural flexibility. Some individuals' flexibility may be limited by certain behavioural and cognitive biases (Mendl et al., 2009), such as neophobia and caution in the presence of novel ambiguous stimuli, which are likely linked with a negative affective state. Moreover, the propensity to influence and be influenced by others' affective states may not be equal among individuals. For example, it may depend on factors such as one's own affective state (Leighton et al., 2010). Despite such predispositions, relatively neophobic individuals may expand their behavioural options and flexibility by gathering social information such that they may approach a novel stimulus provided they have learned from other individuals that the stimulus is safe. For example, in wild jackdaws, a corvid species demonstrating high levels of neophobia, risk‐taking behaviour towards novel anthropogenic stimuli was contagious, that is, dependent on the behaviour of others (Greggor et al., 2016). When they encounter novel foods or objects, jackdaws often exhibit wariness, with stereotyped ‘fear hops’ and other behaviours potentially reflecting negative affective states such as anxiety. However, if they observe others interacting with the novel stimuli (potentially without the occurrence of such fear signals), these fear responses are reduced, allowing them to approach and sample the novel stimulus. Therefore, using available social information about others' behaviour and affective states could be particularly adaptive because it allows animals to adjust their knowledge about ambiguous stimuli: thereby facilitating the avoidance of danger and utilisation of new opportunities.

Social information use of animals may vary in the level of flexibility across the lifespan, for instance due to early‐life experiences (Farine et al., 2015). This flexibility may allow animals to use social information more strategically depending on different environmental conditions and past experience (Laland, 2004). For example, some animals may maintain a high level of flexibility throughout their lives, allowing them to switch strategies if conditions change in the short term. By contrast, individuals may also experience a critical period of flexibility during a certain developmental stage, for example, early in life, that may shape their responses in the long term. Conditions experienced in early life impacting their physiology, affective states, cognition and behaviour may thus have short‐term or potentially long‐lasting effects on social behaviour (Boogert et al., 2014) and social information use (Farine et al., 2015), which may constrain flexibility later in life. For example, zebra finch fledglings that were exposed to an experimental treatment increasing their stress hormone levels were less likely to use social information from their parents than juveniles in a control condition (Farine et al., 2015). Therefore, an early‐life physiological difference, which may be associated with an altered affective state, could serve as a developmental cue eliciting adaptive behavioural shifts, such as changes in social learning strategies. Higher levels of stress hormones in offspring may be linked to insufficient parental provisioning (Greggor et al., 2017), potentially corresponding to suboptimal information parents might have about the current environment. This in turn could make a shift in social associations and social information use by offspring adaptive. When faced with human‐induced rapid environmental change, animals may be more likely to rely on such shifts in social information use.

Animals may also use social information about affective states flexibly depending on the socio‐ecological context, such as different aspects of human‐altered environments or different aspects of their social environment. For example, individuals may have accurate personal information about novel anthropogenic food, but may be more uncertain about potential anthropogenic threats, thus relying more on social information in the latter context. Alternatively, reliance on social information use may vary seasonally, as seen in the study on jackdaws discussed above (Greggor et al., 2016). Individuals may also be flexible in their use of social information about others' affective states depending on the type and quality of their social relationships. For example, hens show marked physiological and behavioural responses to behavioural indicators of affective state in their chicks (Edgar et al., 2011), but not to those from familiar adult conspecifics (Edgar, Paul, et al., 2012). This may indicate constrained flexibility in responding to social information from less closely bonded individuals. For example, when faced with an ambiguous anthropogenic stimulus, an individual's response may be influenced more by the affective state of a closely bonded partner than by the affective states of other individuals. Flexibility in social information use may also be advantageous if some social partners provide more reliable information than others, and individuals may thus benefit from discriminating between different social partners when using social information (social learning strategies: Laland, 2004).

Individual variation in flexible social information use could have fitness consequences because being responsive to others' affective states may only be adaptive in certain contexts and may in fact be maladaptive in others. It is well understood that, despite being less costly to obtain than individually acquired information, social learning is only adaptive if it is strategic or targeted, allowing animals to avoid acquiring socially transmitted information that is outdated, irrelevant or dangerous (Giraldeau et al., 2002). Animals can achieve such targeted information acquisition by employing social learning strategies such as ‘when’ and ‘who’ strategies that allow them to learn only under specific circumstances (such as when unsuccessful) and from certain individuals (such as successful individuals) (Laland, 2004). Similarly, animals may also be expected to be selective, and potentially flexible, in how susceptible they are to emotional contagion. For instance, being unresponsive to others' affective states could be maladaptive if it means that an individual does not acquire information about the presence of a potential predator (i.e. a ‘false‐negative’ response). Conversely, unselectively acquiring the affective states of others, regardless of their characteristics or identity, could also be maladaptive (e.g. in the case of ‘false positives’). For instance, it is conceivable that associating with stressed or ‘pessimistic’ individuals (leading to emotional contagion) may cause one to inappropriately assess risk, resulting in lost opportunities (Brandl et al., 2022). We should therefore expect the affective states of some individuals to be more influential than others and that individuals will vary in their susceptibility to being influenced by others' affective states. For example, one could hypothesise that experience‐ and age‐dependent differences exist in terms of the susceptibility to (being influenced by) false alarms. This is seen in vervet monkeys (*Chlorocebus pygerhythrus*), where infants are less discriminative in their alarm responses than adults, but infant responses become more adult‐like in the presence of their mothers (Seyfarth & Cheney, 1980).

### 2.4 Value and importance of social information use about affective states

As we have highlighted, the use of social information about affective states can influence an individual's well‐being (synonymous with welfare, of which affective state is a key determinant) and fitness outcomes (i.e. survival and reproduction), and as such, it has applied welfare and conservation consequences. For example, assessing affective states could provide a valuable indicator of a population's overall health by identifying negative effects of anthropogenic stressors through more nuanced information than measures such as morbidity and mortality. Developing our understanding of how affective states are propagated and buffered can also help to identify species whose social structure may leave them particularly vulnerable or resistant to anthropogenic change: allowing for more targeted welfare and conservation measures. For instance, species which form strict dyads such as pair‐bonds may be more susceptible to emotional contagion from their partner than species that form loose associations in larger groups. Conversely, forming strong social bonds may increase resilience by facilitating social buffering. Despite its importance, there remains a paucity of interest in affective states within the field of conservation. We consider this issue in Section 3 and discuss the applied value of considering welfare in the context of conservation.

## 3 APPLIED CONSEQUENCES AND IMPLICATIONS

To date, our concern for the well‐being of animals has predominantly focused on those under human control (such as companion, agricultural and zoo animals) because of a widespread opinion that wild animals are not our responsibility (Brakes, 2019). While this may seem reasonable because we are less directly involved in influencing their lives, and because they are affected by many other factors beyond our control such as predation and competition between conspecifics, human‐induced environmental change is now so profound that many wild species are affected by our actions. We therefore have a moral responsibility to better understand our impact on their health and welfare (Brakes, 2019). There are various philosophical and ethical frameworks through which the impact of human activities on wild animal welfare could be evaluated to inform appropriate actions. For example, utilitarian views, which are grounded in the assumption that actions should be evaluated based on their consequences, advocate maximising greater good and minimising harm. Assuming that suffering is an important harm (and its prevention is the greater good), this view could extend to all sentient beings, including animals (for a discussion of sentience, see Browning & Birch, 2022). According to this framework, harming sentient beings, for example, in biomedical research, is acceptable provided the total benefits (e.g. reducing human disease and suffering) outweigh the harms. Under this view, animal welfare is important but may be compromised if conflicting with other goals. For instance, in the case of human–wildlife conflict, the use of deterrents (e.g. guard dogs) to control encounters between foxes (*Vulpes vulpes*) and livestock may seem preferable to lethal measures but can still compromise the welfare of individuals (e.g. hunger from displacement or injuries from deterrents) (de Ridr & Knight, 2024). Another trade‐off arises when a very effective method for controlling a population causes welfare harm while another method prioritising individual welfare could be less effective or more logistically challenging (e.g. poisoning or shooting instead of live trapping and subsequent relocation, and even the latter may have welfare consequences) (Reynolds, 2004). By contrast, deontological frameworks take a stronger, more abolitionist view, arguing that animals have inherent rights that we have the duty to defend, and that this duty cannot be overridden by specific interests and circumstances. Although some frameworks posit this duty extends to all wild animals, others consider that welfare concerns are mainly pertinent in the context of improving conservation outcomes: for example, interventions aimed at reversing or slowing population decline (Beausoleil et al., 2018; Hecht, 2021). Indeed, current research focuses on the global benefits and costs of anthropogenic change, using metrics such as reproductive success, species abundance or distribution and density to gauge stability and resilience of populations (Akçakaya et al., 2018; Harvey et al., 2020). However, while they may well correlate (Greggor et al., 2018), one must not confuse stable populations with positive welfare because it is possible to survive and reproduce while in a negative affective state (Harvey et al., 2020).

Considering wild animal welfare is of particular importance because, as we have discussed, salient information about affective states can influence an individual's resilience and vulnerability to environmental change (Brakes, 2019). Recent efforts to bridge the gap between global‐ or population‐scale conservation approaches and the individual focus of animal welfare concerns through ‘conservation welfare’ promise a pragmatic way forward (Beausoleil et al., 2018). Harvey et al. (2020) propose a framework to integrate welfare considerations into conservation strategies, tailoring interventions to the specific biotic and abiotic needs of species. In the context of reintroduction and relocation programs, scholars such as Miller et al. (2022) and Logan et al. (2023) promote a more hands‐on approach, involving enrichment (i.e. ‘identifying and providing the environmental stimuli necessary for optimal psychological and physiological wellbeing’; Reading et al., 2013) which has long been a tool used to enhance the welfare of captive animals (e.g. Newberry, 1995; Young, 2003). By combining enrichment with the selection of individuals exhibiting behavioural temperaments or cognitive profiles better suited to the wild environment (e.g. fast learners or those with lower neophobic responses), animals may not only be better cognitively equipped to handle environmental challenges and opportunities upon release but may also experience a more positive affective state by reducing fear and distress. These challenges, for example predation, can occur relatively quickly after the introduction in their new environment. When introduced in a wild environment, individuals are often unable to cope with predation risk as they are unable to recognise and act upon it (Reading et al., 2013). By preparing captive animals to detect and avoid predators, enrichment methods can help reduce unnecessary stress and provide the adequate cognitive tools that will facilitate predation recognition. Methods that enhance enrichment and that could prepare individuals for reintroduction into the wild have, for instance, been used by Miller et al. (1990) who confronted captive‐raised Siberian polecats (*Mustela eversmanni*) with a remotely controlled stuffed owl and badger combined with a mild aversive stimulus. The polecats showed an increase in alert behaviour after one single attack. Introducing individuals that are better prepared for the environment could provide social support, enabling conspecifics to interpret affective states more effectively and adapt more successfully to human‐altered environments.

Evaluating the effectiveness of measures such as these is essential. While conservation biology typically assesses success through long‐term population indicators, assessment of individual welfare offers a complementary and more immediate evaluation metric. As we have seen, although affective states cannot be directly measured, they can be inferred from behavioural, physiological and cognitive indicators. For instance, thermal imaging has been used to successfully detect physiological stress in wild birds and mammals. This method (which detects reductions in surface body temperature caused by the sympathetic nervous system directing blood to the core during stress) is non‐invasive and rapid: changes can be detected in as little as 10 s (Jerem et al., 2015). Although there are some methodological challenges for its use in nature (such as controlling for ambient temperature), thermal imaging promises to provide a valuable insight into affective responses to novel stimuli, social contagion and social buffering in the wild. As discussed earlier, cognitive techniques such as the measurement of judgement bias have been used to assess affective states in many captive animals including rodents, dogs, primates, dolphins, fish (Burman et al., 2011; Clegg et al., 2017; Lagisz et al., 2020; Neville et al., 2020) and even insects (but note: whether insects experience affective states remains contentious—see Barron & Klein, 2016; Key et al., 2016), and recently, an ingenious approach has been used to measure this indicator in free‐living fish (Freire & Nicol, 2024). Using the fish's natural attraction to light, their avoidance of predators, and the following stimuli: (a) positive stimulus—light‐only; (b) negative stimulus—light and large predator model; (c) ambiguous stimulus—light and small predator model; the authors were able to run the task without training. They evaluated the number of fish attracted to the different stimuli, and how attraction to the ambiguous stimulus was related to aspects of water quality that may influence fish health and associated affective state (Freire & Nicol, 2024). The study found that fish approached the positive stimulus more than the negative, while there was greater avoidance of the ambiguous stimuli as water quality decreased (increased salinity and phosphorus, and lower pH): indicating a negative population‐level judgement bias (Freire & Nicol, 2024). By measuring how individuals' affective states are impacted by environmental changes such as an increase in water turbidity, the evaluation of anthropogenic activities' impact is becoming more efficient and can drastically decrease the large‐scale negative consequences over populations or even ecosystems.

Not only can these methods allow us to measure affective states in the wild but also their results can provide the information necessary for improving existing welfare interventions. For example, providing supplemental bird feeders in residential gardens is such a popular pastime that it is now a multibillion‐dollar industry (Plummer et al., 2019). Although this can be positive for conservation because it can improve the physiological health of individuals, increase local bird populations and engage people with nature (Cox & Gaston, 2016; Plummer et al., 2019; Wilcoxen et al., 2015), little is understood about its impact on individual affective states. For instance, the design of feeders could inadvertently increase stress if they do not allow for social support and buffering. Similarly, the installation of nestboxes has generally proved to be an effective conservation method, particularly in human‐altered environments. For example, breeding numbers of storm petrels (*Hydrobates pelagicus melitensis*) on Benidorm Island, which dwindled due to habitat deterioration, increased greatly following the installation of nestboxes (Libois et al., 2012). However, nestboxes in high densities could negatively impact affective states by intensifying competition, aggression and stress. This seldom considered welfare concern deserves greater investigation, not least because positive affective states have been linked to improved longevity, health and reproductive fitness. Indeed, self‐reported ‘happy’ humans live longer and suffer less morbidity (Diener & Chan, 2011), and negative affective states may also be linked to morbidity and mortality in other animals (see Walker et al. (2012) for a review). For example, domestic dogs (*Canis lupus familiaris*) that exhibited very pronounced fear of strangers lived shorter lives, which may indicate a potential impact of negative affective states on health and longevity. This link also suggests that affective states could be important indicators of and causal factors for the resilience of wild populations in response to anthropogenic change.

## 4 CONCLUSION AND FUTURE DIRECTIONS

In this paper, we aimed to synthesise current knowledge on how (wild) animals use their own and others' affective states to cope with human‐induced environmental change and highlight important gaps in our understanding. There is still a dearth of research on affective states in wild animals, and their role as sources of social information in response to human‐altered environments remains largely unexplored. Research on captive animals suggests that emotional contagion may be widespread and provides tools to determine the impacts of housing and husbandry on welfare, providing an evidence base for effective interventions. However, little attention has been given to the assessment of affective states in wild animals as a way of evaluating the impact of human‐induced environmental change on their welfare and establishing links between these states and the ability to survive and reproduce. Bridging fundamental research on animal affective states with applied approaches in welfare and conservation will be essential to addressing this knowledge gap. Additionally, technological advances currently used to assess affective states in captive animals could be adapted for wildlife populations, providing novel insights into their welfare and potential to respond adaptively to anthropogenic pressures. Given the growing influence of human activities on natural ecosystems, we strongly encourage future research to prioritise this topic. A deeper understanding of affective states in wildlife will be instrumental in developing more effective conservation strategies that account for both population dynamics and individual well‐being.

### Glossary

**Table:** 

Term	Definition	References
Affective states	Valanced (that is, positive or negative) mental states which consist of short‐term emotions and longer‐term moods. Emotions last from seconds to minutes and are caused by a specific event, whilst moods are ‘free floating’ states not linked to any specific event.	Mendl and Paul (2020); Rault et al. (2025)
Appraisal	Inherently transactional process between the individual and the environment, in which the significance of the event must be detected and assessed by the appraiser. Appraisal components allow the evaluation of an event, by combining both the individual's affective state and the momentary environmental conditions as contributing factors to the appraisal process.	Faustino et al. (2015)
Behavioural flexibility	The ability to modify behaviour in response to changing conditions, a crucial strategy for coping with anthropogenic impacts.	Wolf et al. (2008)
Distress	Negative affective state resulting from a stimulus for which the animal has no adaptive response.	Reading et al. (2013)
Emotional contagion	The matching of perceived affective states among conspecifics. In other words, an individual shifts their own affective state in the same direction as another's.	Meyza et al. (2017); Pérez‐Manrique and Gomila (2022); Špinka (2012)
Empathy	The capacity to be affected by, and share, the perceived (invariably negative) affective state of another individual.	De Waal (2008); Preston and de Waal (2002)
Human‐induced environmental change	Refers to the alterations in the natural environment that are primarily caused by human activities. These changes can include various factors such as urbanisation, pollution, climate change, habitat destruction, and the introduction of non‐native species.	Mazza et al. (2020); Sih (2013)
Judgement bias	Based on insights from human psychology which reveal that alterations in the way one processes information (known as a ‘cognitive bias’) can be an indicator of whether a person perceives a stimulus as positive or negative. One such cognitive bias is ‘judgement bias’: whereby self‐reported ‘happy people’ respond more ‘optimistically’ to an ambiguous stimulus than ‘pessimists’ who suffer negative affective states. Studies on a range of species have revealed similar trends, and subsequently, the judgement bias task is considered the most validated method of assessing affective states in non‐human animals.	Appleby et al. (2018)
Social information	Information obtained from observing and tracking others' behaviour and interactions with the environment. Social information can be acquired from and about others.	Danchin et al. (2004)
Stress response	A physiological response to external stimuli that are perceived as stressor. Can involve changes in neural and hormonal activity that induce shifts in metabolism to ensure the maintenance of vital functions and the mobilisation of vital resources.	Sapolsky et al. (2000)
Uncertainty	A concept from information theory. The probability with which a prediction can be made given available information. Uncertainty is high when different outcomes of a parameter are equally likely.	Shannon (1948)
Urban adapter	Refers to a species that has is able to occupy urban environments but can utilise both natural and artificial resources.	Shochat et al. (2006)
Urban exploiter	Refers to species that thrive in urban environments and become dependent on anthropogenic resources.	Shochat et al. (2006)
Welfare/wellbeing	Terms are used interchangeably to describe the quality of an animal's subjective experiences.	Rault et al. (2025)

## AUTHOR CONTRIBUTIONS

L.G.H., J.M., M.V. and A.T. conceived the ideas. L.G.H., J.M. and M.V. led the writing of the manuscript. All authors contributed critically to the drafts and gave final approval for publication.

## FUNDING INFORMATION

L.G.H. was supported by the Biotechnology and Biological Sciences Research Council‐funded South West Biosciences Doctoral Training Partnership (DTP3: BB/T008741/1). J.M. is supported by Wild Animal Initiative grant to A.T. (C‐2023‐00054). A.T. also acknowledges the generous support of the Leverhulme Trust (grant RGP‐2020‐170).

## CONFLICT OF INTEREST STATEMENT

The authors have no conflicts of interest to declare.

## ETHICS STATEMENT

No ethics approval was required for this paper.

## AI STATEMENT

AI technologies have been used according to the code of conduct in the preparation of this paper regarding the assistance for correct transcription from French.

## Data Availability

None.
